# Investigating the dynamic responses of *Aegilops tauschii* Coss. to salinity, drought, and nitrogen stress: a comprehensive study of competitive growth and biochemical and molecular pathways

**DOI:** 10.3389/fpls.2023.1238704

**Published:** 2023-09-06

**Authors:** Rashida Hameed, Adeel Abbas, Muhammad Saeed, Aitezaz A. A. Shahani, Ping Huang, Daolin Du, Usman Zulfiqar, Saud Alamri, Alanoud T. Alfagham

**Affiliations:** ^1^ Institute of Environment and Ecology, School of Environment and Safety Engineering, Jiangsu University, Zhenjiang, China; ^2^ Department of Weed Science and Botany, The University of Agriculture, Peshawar, Pakistan; ^3^ Key Laboratory of Crop Sciences and Plant Breeding Genetics, College of Agriculture, Yanbian University, Yanji, Jilin, China; ^4^ Department of Agronomy, Faculty of Agriculture and Environment, The Islamia University of Bahawalpur, Bahawalpur, Pakistan; ^5^ Department of Botany and Microbiology, College of Science, King Saud University, Riyadh, Saudi Arabia

**Keywords:** abiotic stress, *Aegilops tauschii* Coss, gene expression, physio-chemical properties, mechanism

## Abstract

*Aegilops tauschii* (Coss.) is a highly deleterious, rapidly proliferating weed within the wheat, and its DD genome composition exhibits adaptability toward diverse abiotic stresses and demonstrates heightened efficacy in nutrient utilization. Current study investigated different variegated impacts of distinct nitrogen concentrations with varied plant densities, scrutinizing the behavior of *Ae. tauschii* under various salinity and drought stress levels through multiple physiological, biochemical, and molecular pathways. Different physiological parameters attaining high growth with different plant density and different nitrogen availability levels increased *Ae. tauschii* dominancy. Conversely, under the duress of salinity and drought, *Ae. tauschii* showcased an enhanced performance through a comprehensive array of physiological and biochemical parameters, including catalase, peroxidase, malondialdehyde, and proline content. Notably, salinity-associated traits such as sodium, potassium, and the sodium–potassium ratio exhibited significant variations and demonstrated remarkable tolerance capabilities. In the domain of molecular pathways, the *HKT* and *DREB* genes have displayed a remarkable upregulation, showcasing a comparatively elevated expression profile in reaction to different levels of salinity and drought-induced stress. Without a doubt, this information will make a substantial contribution to the understanding of the fundamental behavioral tendencies and the efficiency of nutrient utilization in *Ae. tauschii*. Moreover, it will offer innovative viewpoints for integrated management, thereby enabling the enhancement of strategies for adept control and alleviation.

## 1 Introduction

*Aegilops tauschii* Coss (*Ae. tauschii*) is classified as a highly invasive and non-native weed, presenting considerable challenges, especially in regions where wheat cultivation is widespread. It is native to temperate and tropical regions of Asia and Europe and exhibits aggressive traits as a weed species in several provinces of the People’s Republic of China (Kim et al., 2021; Yang et al., 2023). Because of deficit of evidence, inadequate prevention, and constraint of controlling strategies, the pace of spreading this weed remarkably damages winter wheat production and development, especially in the major wheat producing regions of P.R. China (Boruah et al., 2023; Singh and Thakur, 2023). This weed actively contests for nutrients [including nitrogen (N)]. Furthermore, its D genome contains an extensive array of genetic material, harboring numerous traits that can be utilized to enhance wheat characteristics through breeding. These traits encompass resilience to drought, salinity, and heat stress (Molero et al., 2023). At present, researchers are actively involved in assessing these favorable traits, and they have effectively pinpointed distinct genes that play a role in enhancing wheat crops. Utilizing gene expression analysis, scientists are striving to attain a more profound comprehension of the molecular mechanisms employed by plants to counteract diverse abiotic stresses for wheat improvement program (Mondal et al., 2023).

In various weed control cultivation methods, adopting an elevated crop seeding rate is a cultural practice for managing weeds. This approach effectively enhances the competitive ability of winter wheat against diverse weed species while also ensuring optimal nutrient availability, including N (Loura et al., 2023; Restuccia and Scavo, 2023). Optimization application of N is an essential role for adjusting crop–weed competitive relationship, and role of higher plant density/narrow row spacing is considered as an important and reported a positive strategy for controlling weeds (Dionisi et al., 2023; Liu et al., 2023). Adjusting plant density association with different N fertilizer supplies is considered as an important component for knowing crop–weed competitive interaction and its application effects different physiological and biochemical activities (Horvath et al., 2023). N fertilizer also showed impact on photosynthesis activities and directly links with chlorophyll activities of plants (Zhang et al., 2023). Therefore, it is imperative to comprehend the intricate relationship between N and abiotic stress in plants to optimize the utilization of N fertilizers. This understanding allows us to strike a delicate balance between N application and mitigating the detrimental impacts of abiotic stresses (Etesami et al., 2023).

The exploration of stress mechanisms centers around understanding how plants perceive stress and the biochemical, physiological, and genetic changes that occur as a result of stress. The study of stress mechanisms delves into how plants perceive stress and the subsequent physiochemical and genetic changes. In bread wheat, the *Ae. tauschii* D genome has been observed to exhibit reduced Na^+^ exclusion, leading to increased sensitivity to salinity (Abbas et al., 2021a; Křížkovská et al., 2023). Salinity tolerance mechanisms, such as the indicative regulation of specific genes like HKT1-4, are evident in various plants, including *Ae. tauschii*. Salinity stress affects plant biochemistry and physiology at the cellular level, as well as on a larger scale involving the entire plant, resulting in both ionic and/or osmotic stresses (Ndecky et al., 2023). The action pathway of stress can produce both immediate and long-term effects on plants. Initially, it influences the rhizosphere and exposed plant parts, whereas its long-term consequences impact the entire plant, depending on the specific stress (Sohrabi et al., 2023). At the cellular level, stress triggers metabolic toxicity, interferes with photosynthesis, generates reactive oxygen species (ROS), disrupts cell membranes, and can even induce programmed cell death (Kang et al., 2023; Liu et al., 2023). In addition, various physiological, biochemical, and molecular changes are observed, including the development of severe necrotic lesions on leaves, reduced growth, a decrease in the number of leaves, and impaired plant reproductive capacity (Hilário et al., 2023; Lungoci et al., 2023).

Among various abiotic stresses, drought condition significantly decreases the relative water content and photosynthesis activities and hampers cell division process. In addition, it disrupts the delicate balance ROS at the antioxidant level (Abdirad et al., 2022). Different plants including weeds undergo different physiochemical and molecular alterations in response to different abiotic stresses, including drought. Different aspects of plant growth, such as chlorophyll content and photosynthetic activity, are directly affected by varying levels of drought stress. Water stress inhibits cell function, retards plant growth, triggers the activation of repair systems, and leads to metabolic changes as part of general adaptive responses (Wang et al., 2022). Understanding plant responses to diverse abiotic (salinity and drought) stresses is crucial for executing effective crop management approaches that accelerate biochemical and molecular activities (Ali et al., 2022). Furthermore, these stresses disrupt the normal physiological processes of plant cells, particularly by affecting the ROS levels (Vijayaraghavareddy et al., 2022).

When plants experience drought stress and the consequent oxidative damage, they employ mechanisms to either circumvent or restore the harmful effects affected by ROS construction (Fadiji et al., 2022). This is achieved through the action of various enzymes, such as catalase (CAT), peroxidase (POD), and superoxide dismutase (SOD), entering the plants. It has been observed that SOD, POD, and CAT activities played an energetic part in maintaining plants from severe condition (Jangir et al., 2021). In addition, in the mechanisms governing drought and salinity stress tolerance, multiple genes are involved, which are associated with morphological and structural expression changes in plants (Manna et al., 2021; Wu et al., 2022). Drought lenience exhibits involved phenotypic and variable attributes, which encompass these rejoinders and comprise numerous molecular ways (Bukhari et al., 2022).

On the basis of the currently available body of published research and the outcomes of our previous experiments (Abbas et al., 2020; Abbas et al., 2021a; Abbas et al., 2021b; Yu et al., 2021), the present research endeavors to propose that modifications in N dosages and plant density of *Ae. tauschii* have an impact on wheat growth by means of a sigmoid model, which assesses the competitive growth of both *Ae. tauschii* and wheat. Furthermore, it is hypothesized that varying degrees of salinity and drought will result in distinct physiological, biochemical, and molecular alterations in *Ae. tauschii*. These alterations will be analyzed to ascertain the effects of salinity and drought on *Ae. tauschii* across different magnitudes, as well as their implications for the plant’s overall growth and development.

## 2 Materials and methods

This experiment involved the utilization of *Ae. tauschii* populations obtained from fiver different provinces in P.R. China. The local wheat variety Ningmai 13 was chosen for experimentation. The seeds used in the study were stored at the herbarium of the Institute of Plant Protection, Chinese Academy of Agricultural Sciences in Beijing, China. For the first hypothesis, plastic parts with size 20 cm × 25 cm were used to investigate different N applications with different plant density levels of *Ae. tauschii* and filled with 1 kg of soil. A factorial design was implemented in this experiment, consisting of two main factors. The main factor involved different N rates, namely, 0 kg ha^−1^, 60 kg ha^−1^, 120 kg ha^−1^, and 180 kg ha^−1^. The sub-factor encompassed various wheat crop densities, specifically 0, 100, 200, and 400 plants m^−2^. The experimental units were replicated three times. To maintain the desired number of wheat and weed plants in each pot, thinning was carried out 10 days after sowing (DAS). N was applied into two equal split doses: one after 3 weeks and the second after 6 weeks of emergence. The wheat plants, at densities of 100, 200, and 400 plants m^−2^, were sown around the *Ae. tauschii* weed, maintaining equal spacing between them.

For the application of salinity and drought stress, the experiment involved planting seeds in plastic pots filled with sand within a controlled greenhouse environment. To ensure proper drainage, excess water was allowed to flow out through holes at the bottom of each pot, collecting on plates positioned underneath. Initially, irrigation was carried out using tap water. After 1 week, when the plants reached the second leaf stage, the tap water was replaced with Hoagland solution. The Hoagland solution was gradually administered in five increments until the final salt concentrations reached 300 mM NaCl, representing three salinity treatments (0, 50, 100, and 200 mM NaCl) with eight replications. The experimental design used for the study was a randomized complete block design with a split arrangement. To induce drought stress, two different concentrations of PEG-6000, specifically 0 g L^−1^ and 75 g L^−1^, were applied in eight replications to evaluate various physiological, biochemical, and molecular parameters.

### 2.1 Physiological and biochemical indices

The following data regarding physiological parameters were collected at various time points, specifically 13, 26, 39, and 52 DAS. For the wheat crop and weed plants, Soil Plant Analysis Development (SPAD) data were recorded using a TYS-3N model 400-672-1817 from China. Leaf area data were obtained with the assistance of a leaf area meter (YMJ-B model 400-672-1817, China). These measurements were taken under different N and plant density levels. The concentrations of Na^+^ and K^+^ were determined using a flame photometer (Shabala et al., 2013). To assess *Ae. tauschii* under field capacity conditions, the plant was placed in a dark room for 30 min. Subsequently, dry weight biomass and plant height of *Ae. tauschii* were recorded under various salinity and drought stress levels. Chlorophyll fluorescence data were gathered utilizing the imaging-PAM Mini-Series (IMAG-K5, Walz, Germany) across various N, salinity, and drought stress levels (Itam et al., 2020). The imaging-PAM mini-series apparatus was employed to assess several parameters, including the minimum fluorescence (Fo′), the maximum fluorescence (F′m), the quantum yield of photosystem II (ΦPSII), and the photochemical quenching (qP). The following formulas were used for qP and qN. The antioxidant defense systems were assessed by measuring the levels of POD, CAT, proline, and MAD under different salinity and drought stress levels (Hajihashemi and Ehsanpour, 2013). The activity of POD was resolute using the guaiacol method. CAT was measured through the UV captivation procedure. Proline content was determined using the acidic ninhydrin method. The content of MAD was assessed using the thiobarbituric acid method (Lungoci et al., 2023). These measurements provided insights into the antioxidant capacity and oxidative stress levels in retort to salinity and drought stress.

### 2.2 RNA extraction, cDNA synthesis, and real-time PCR analysis

Genomic DNA extraction was carried out using the RNase-free DNase1 kit (cat. no. DP432, Tiangen Biotech Beijing Co., Ltd., Beijing, China) from 1 g of leaf sample. The quality of the RNA and DNA was evaluated using 1% agarose gel electrophoresis, whereas the quantity and quality of RNA were assessed using a NanoDrop (NanoDrop Technologies, Wilmington, DE, USA). For cDNA synthesis, 1 μg of RNA was utilized with the fast quant R.T. kit (cat. no. KR116-02, Tiangen Biotechnology Co., Ltd., Beijing, China). quantitative polymerase chain reaction (qPCR) analysis was performed using an ABI 7500 machine and SYBR green (Applied Biosystems, Foster City, CA, USA). Each sample had a total volume of 20 μL, consisting of 10 μL of PCR master mix, 0.6 μL of forward and reverse primers, 0.6 μL of dye, and 7.3 μL of ddH_2_O. The cycling conditions for the melting curve temperature were as follows: an initial 10-min incubation at 95°C, followed by 40 cycles of denaturation at 95°C for 5 s, annealing at 55°C–58°C for 32 s, and extension at 72°C for 30 s. The melting curve temperature was increased by 0.5°C to 0.7°C every 5 s. The qPCR analysis was conducted with four biological replicates and three technical replicates. Gene sequences were obtained from the National Center for Biotechnology database for primer design. Forward and reverse primers (5′-3′) of reference genes were designed by Beacon software (**Supplementary Table 1**).

### 2.3 Data analysis

The statistical analysis of physiological traits and salinity tolerance traits (Na^+^, K^+^, and K^+^/Na^+^) was conducted using SAS version 9.3. The means of the treatments were separated using the standard error of differences at a significance level of 5% under varying salinity and drought stress levels. The data pertaining to different parameters of *Ae. tauschii* of different physiological parameters (plant height, number of tillers per plant, and number of leaves per plant) were fitted to a three-parameter sigmoid model. This modeling approach helped to analyze and understand the growth and development patterns of *Ae. tauschii* in response to the experimental conditions. In the aforementioned model, Y represents the assessed constraints at time x. The parameter “a” represents the maximum value, and “x50” denotes the time required to reach 50% of the parameter value, such as the plant height, the number of tillers per plant, or the number of leaves per plant. The parameter “b” indicates the rate of change for the different parameters. For the analysis of leaf and inflorescence biomass data, an exponential decay model with two parameters was employed. The data for various parameters of *Ae. tauschii*, including leaf area, stem biomass, and whole plant biomass, were fitted to a two-parameter sigmoid model. In the sigmoid model, “y” represents the estimated parameter resulting from different weed planting densities, N rates, or DAS. The parameter “a” represents the lowest parameter value at the maximum wheat planting density of 440 plants m^−2^, and “y0 + a” represents the highest parameter value at the minimum wheat planting density of 0 plants m^−2^. The parameter “b” represents the slope of the sigmoid curve.

## 3 Results

### 3.1 Morpho-physiological parameters under different nitrogen levels with plant density

Increasing plant density from 110 to 440 plants m^−2^ affected the height of *Ae. tauschii*, with a sigmoid model predicting a maximum height of 54 cm when grown alone (**Table 1**). At a density of 440 plants m^−2^, the attained height was only 20 cm, and increasing plant density resulted in a decrease in height. However, plant height increased up to 390%. The time essential to influence 50% of the plant height decreased as the planting densities increased, and the application of different N rates also had an impact on plant height. Higher N levels ensued in a significant expand in weed height. The growth response of *Ae. tauschii* in terms of the number of leaf and the number of tillers per plant exhibited a sigmoidal pattern (**Table 2**). *Ae. tauschii* was projected to have seven tillers per plant at a weed density of 0 plants m^−2^ in a wheat crop, but this number decreased as the weed density increased. The number of tillers in wheat increased with plant density, but the model did not work for the highest density. At 52 DAS, wheat plant densities decreased the number of leaves by 65%–81%, with a significant increase in leaf number observed with increasing N rate. These findings can inform wheat crop management practices to optimize yield by managing weed density and using appropriate N rates.

**Table 1:** Growth prediction model between *Ae. tauschii* and wheat through different physiological parameters under different nitrogen and plant density levels.

Plant density	Plant height				Number of leaves				No. of tillers			
	A	B	x0	R^2^	a	b	x0	R^2^	a	B	x0	R^2^
0	54.39 (4.34)	15.99 (2.02)	46.55 (2.57)	0.99	23.51 (0.50)	9.73 (0.34)	35.15 (0.58)	0.99	7.00 (0.08)	6.82 (0.39)	18.20 (0.40)	0.99
110	34.47 (4.66)	15.67 (3.61)	39.49 (4.88)	0.99	7.91 (0.08)	10.02 (0.31)	23.38 (0.35)	0.99	3.00 (0.34)	0.60 (0.09)	12.65 (1.53))	0.99
220	23.71 (1.55)	15.31 (0.80)	30.55 (2.31)	0.99	4.83 (0.31)	6.40 (2.46)	15.12 (2.07)	0.98	1.96 (1.31)	0.66 (0.02)	11.42 (1.51)	0.99
440	20.42 (3.01)	14.16 (1.42)	29.07 (5.59)	0.99	4.23 (0.14)	9.89 (1.58)	14.26 (1.08)	0.98	Model not fitted			
Nitrogen levels												
0	17.84 (0.33)	13.79 (3.09)	29.94 (0.61)	0.99	2.01 (0.20)	8.62 (0.34)	1.34 (0.43)	0.99	4.20 (0.29)	12.15 (2.92)	18.08 (2.27)	0.98
40	27.12 (4.78)	12.56 (0.40)	31.75 (1.32)	0.98	2.90 (0.05)	4.31 (0.31)	11.73 (1.25)	0.99	8.11 (0.38)	12.71 (1.01)	30.27 (1.63)	0.99
80	29.49 (1.25)	11.54 (0.82)	34.64 (5.94)	0.99	3.43 (1.01)	6.57 (1.46)	13.13 (2.47)	0.99	12.44 (1.79)	12.59 (2.37)	34.73 (4.67)	0.98
120	31.66 (1.59)	10.87 (1.00)	30.88 (1.52)	0.99	4.74 (1.12)	6.02 (1.28)	15.78 (2.08)	0.99	25.79 (3.64)	12.93 (1.42)	43.56 (4.09)	0.99

A sigmoid growth model, y = (y0 + a)e − bx, was fitted to leaf stem, inflorescence, and total plant dry weight of Ae. tauschii, where y is the stem dry weight and total plant weight at various wheat densities (0–440 plants m^−2^), y0 represents the value at wheat planting densities plants m^−2^, and (y0 +a) represents the highest value at weight planting density.

**Table 2:** Growth prediction model between *Ae. tauschii* and wheat through different physiological parameters under different nitrogen levels.

Nitrogen Level	Leaf dry weight			Stem dry weight				Inflorescence Weight			Plant weight biomass			
	a	b	R^2^	y^0^	a	b	R^2^	A	b	R^2^	y^0^	a	b	R^2^
**0**	1.991 (0.031)	0.005 (0.002)	0.98	0.552 (0.086)	1.452 (0.119)	0.011 (0.002)	0.99	3.948 (0.667)	0.011 (0.004)	0.98	1.660 (0.155)	6.334 (0.231)	0.014 (0.001)	0.99
**60**	2.197 (0.014)	0.004 (6.588)	0.99	1.075 (0.114)	4.921 (0.177)	0.017 (0.002)	0.99	7.698 (0.800)	0.009 (0.002)	0.98	2.527 (0.335)	13.459 (0.489)	0.013 (0.001)	0.99
**120**	2.549 (0.026)	0.004 (0.001)	0.99	1.639 (0.080)	5.364 (0.115)	0.012 (0.008)	0.99	8.972 (0.361)	0.004 (0.001)	0.99	3.065 (0.116)	15.574 (0.139)	0.007 (0.002)	0.99
**180**	2.763 (0.033)	0.004 (0.001)	0.99	1.921 (0.110)	6.333 (0.158)	0.012 (0.009)	0.99	10.794 (0.155)	0.002 (0.001)	0.99	4.014 (0.587)	17.664 (0.611)	0.005 (0.002)	0.99

A sigmoid growth model, y = (y0 + a)e − bx, was fitted to leaf stem, inflorescence, and total plant dry weight of Ae. tauschii, where y is the stem dry weight and total plant weight at various wheat densities (0–440 plants m^−2^), y0 represents the value at wheat planting densities plants m^−2^, and (y0 +a) represents the highest value at weight planting density.

**Figure 1:**
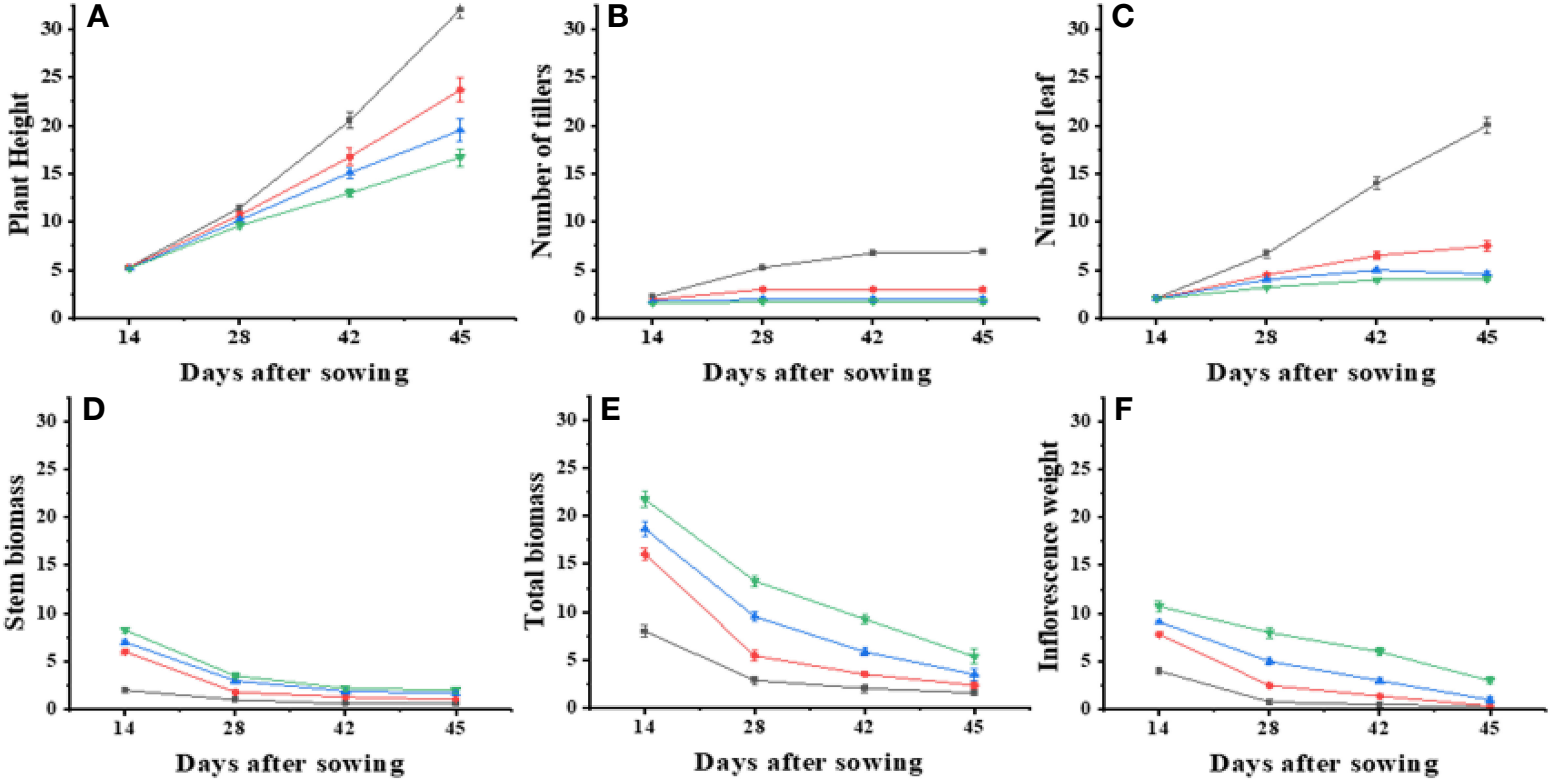
Effect on different physiological parameters under different nitrogen levels with different levels of density. Where’s, **(A)** plant height, **(B)** number of tillers, **(C)** number of leaf, **(D)** stem biomass, **(E)** total biomass and **(F)** inflorescence weight.

**Figure 2:**
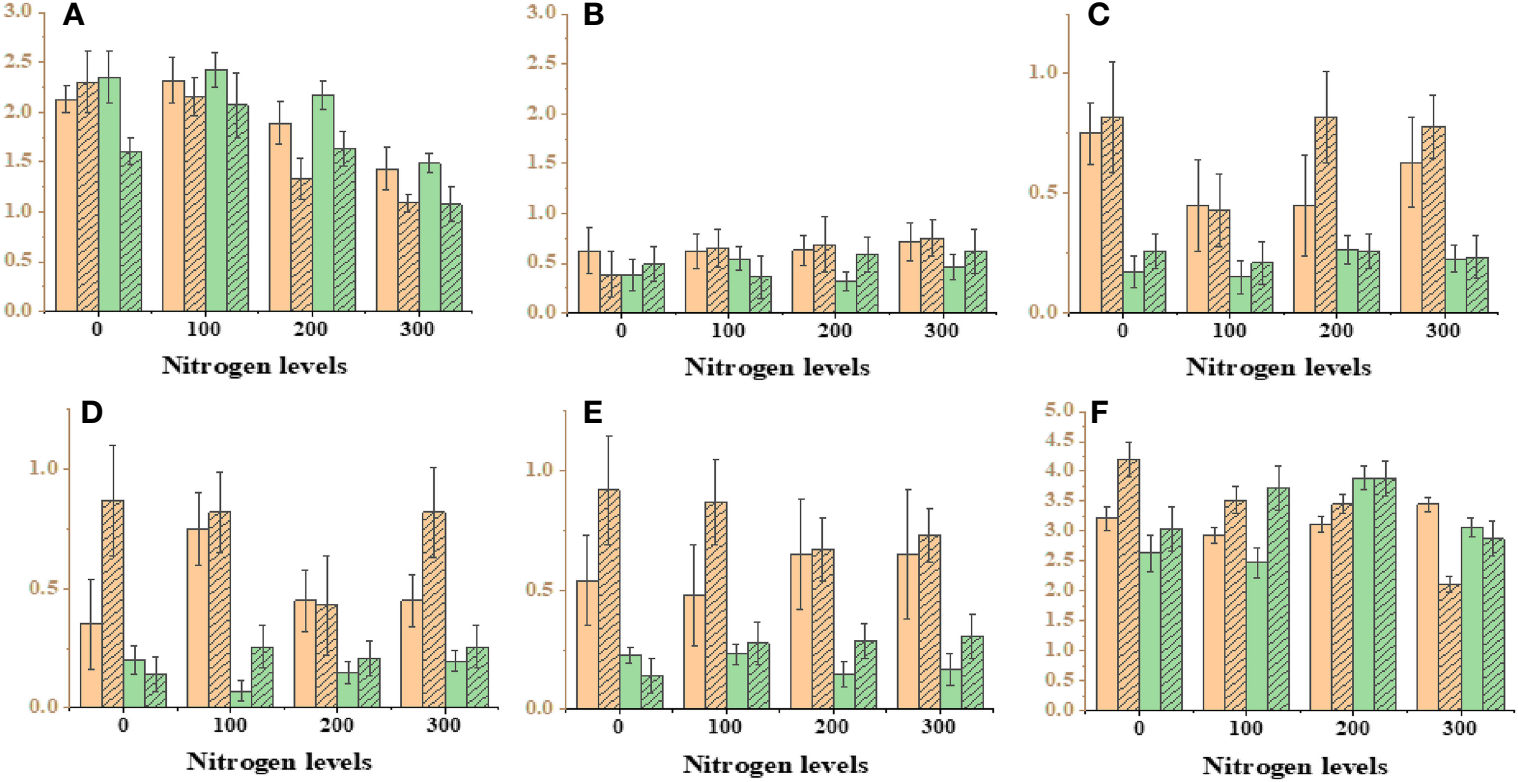
Effect on different photosynthesis parameters under different nitrogen levels with different levels of density. Where’s, **(A)** minimal fluorescence (F0), **(B)** maximal fluorescence (Fm), **(C)** photo chemical quenching (NPQ), **(D)** non-photo chemical quenching (YNPQ), **(E)** inhibition (Inh.1) and **(F)** photosystem second Y(II).

In this study, an exponential model with three parameters was used to analyze the effect of varying N levels and wheat planting densities on leaf area, leaf biomass, and stem dry weight of *Ae. tauschii*. The results showed that wheat planting densities significantly affected leaf area, with the highest density of 440 plants m^−2^, reducing leaf area to 67%–81%, compared with density of 0 plants m^−2^. Application of N levels at 60–180 kg ha^−1^ significantly increased leaf area to 149%–339% compared with N levels at 0 kg ha^−1^. Leaf biomass was highest at the maximum N level and increased by 17%–48% with increasing N levels. Stem dry weight was also significantly affected by wheat planting densities, with the highest density 90-96% reducing stem weight to 67%–81% compared with density of 0 plants m^−2^. These results provide valuable information for optimizing N and planting density management in *Ae. tauschii* cultivation. Current experiments investigated the effects of weed plant density and N levels on various growth parameters of *Ae. tauschii* and wheat crop. Results showed that increasing weed plant density levels meaningfully decreased inflorescence dry weight and total plant weight of *Ae. tauschii*, whereas increasing N levels significantly increased these parameters. In addition, increasing wheat crop planting density also significantly reduced total plant weight and increased leaf weight ratio but decreased the root–shoot ratio of both *Ae. tauschii* and wheat crop (**Supplementary Table 2**). Furthermore, the application of different levels of N significantly increased the SPAD values of both *Ae. tauschii* and wheat crop. The highest SPAD value was observed after 39 days of sowing. These findings suggest that managing weed density and N levels can greatly influence the growth and development of *Ae. tauschii* and wheat crop and may have implications for crop management strategies to improve yield and quality. *Ae. tauschii*–specific stem length (SSL) was affected by increasing weed plant densities (0–440 plants m^−2^) and N levels (0 kg ha^−1^, 60 kg ha^−1^, 120 kg ha^−1^, and 180 kg ha^−1^). SSL was increased by increasing weed plant densities but decreased by increasing N levels. Moreover, SSL was significantly affected by increasing wheat planting densities (110, 220, and 440 plants m^−2^), with a corresponding increase in SSL of 35%, 104%, and 111%, respectively, compared with wheat planting density of 0 plants m^−2^. Conversely, application of N levels at 60 kg ha^−1^, 120 kg ha^−1^, and 180 kg ha^−1^ significantly decreased SSL compared with N levels at 0 kg ha^−1^ (25 cm g^−1^) (**Figure 1**). Data regarding photosynthesis activities, minimal fluorescence (F_0_), maximal fluorescence (Fm), photo (NPQ), and non-photo (YNPQ) chemical quenching and photosynthesis activities showed a positive impact and increased with N and plant density combinations (**Figure 2**; **Supplementary Table 1**).

### 3.2 Effect of different salinity concentrations on physiological and biochemical activities

**Figure 3:**
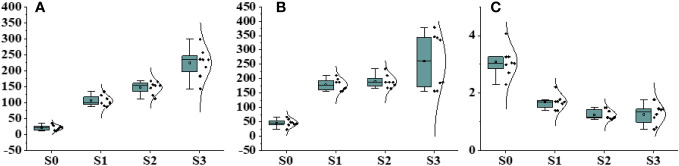
Effect of different salinity stress levels on different physiological parameters. **(A)** Na^+^, **(B)** K^+^, and **(C)** K^+^/Na^+^.

**Figure 4:**
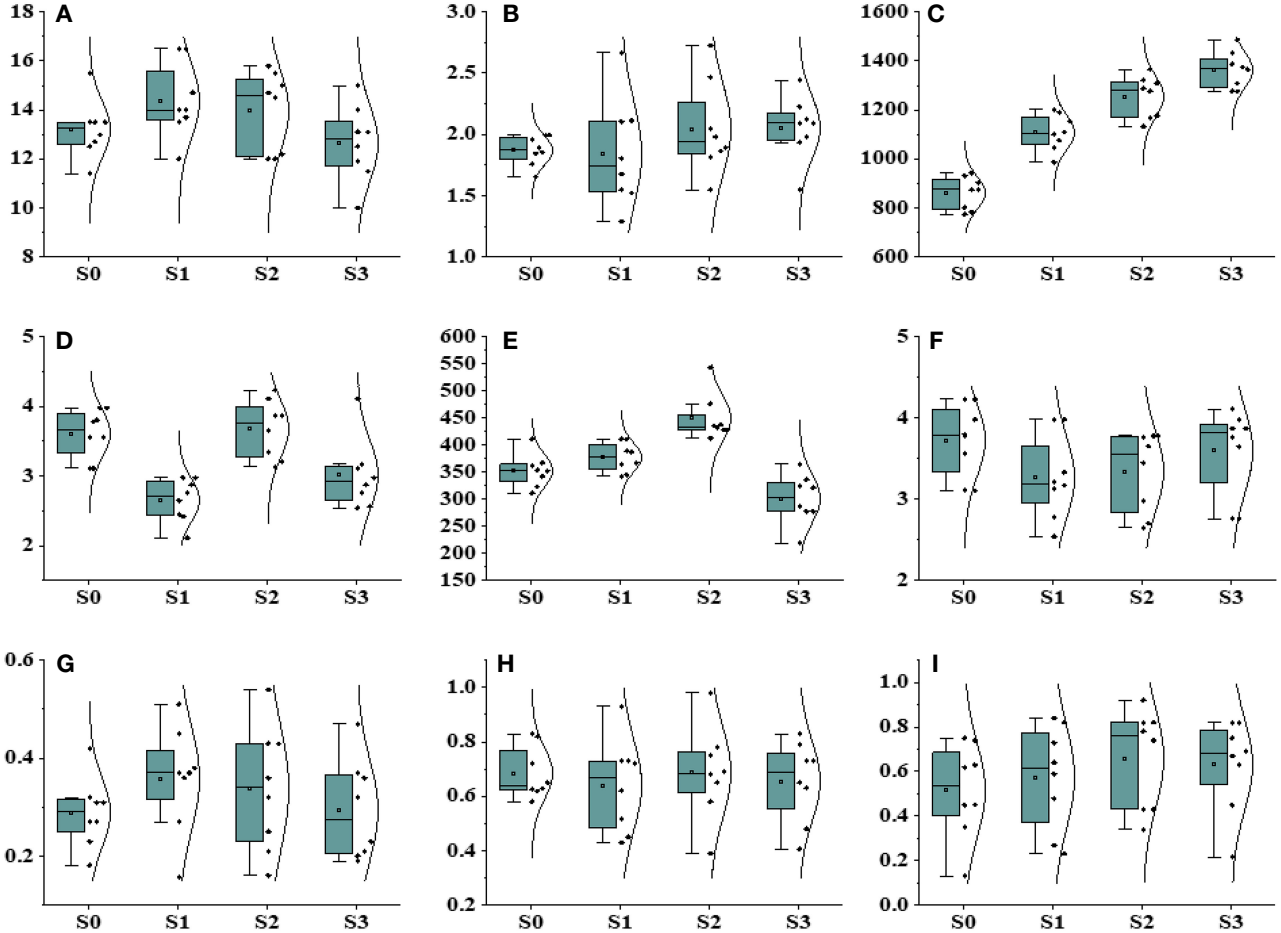
Effect of different salinity levels on different physiological, biochemical, and photosynthesis parameters. **(A)** Plant height, **(B)** plant biomass, **(C)** POD, **(D)** MDA, **(E)** CAT, **(F)** proline, **(G)** maximum fluorescence (FM), **(H)** Quantum yield of regulated energy dissipation, (photochemical quenching) (Y(NPQ)), and **(I)** Inhibition (inh).

Physiological and biochemical parameters were significantly influenced by different levels of salinity. The statistical analysis conducted on different physiological parameters revealed significant differences under different salinity stress conditions and the control group (**Figure 3**; **Supplementary Table 3**). Notable variations were detected in sodium and potassium growth under different salinity levels, with higher salinity levels leading to increased concentrations of sodium and potassium. This accumulation had a direct effect on plant biomass. The influence of salinity on salinity-related parameters became more prominent as the salinity concentration increased. The Na^+^ concentration in individuals exposed to salinity stress treatments was four times lower than the mean value observed in other populations subjected to control treatments. Significant effects on photosynthesis activity, specifically chlorophyll fluorescence, were observed under different salinity stress levels (**Figure 4**). The Fm was recorded at 0.54 in the presence of 200 mM NaCl, whereas the minimum fluorescence of 0.12 was observed under control conditions. Regarding Y(NPQ), an increase was observed under 200 mM NaCl, reaching a maximum of 0.98 and gradually decreasing with decreasing salinity levels (**Supplementary Table 4**). The proliferation in non-photochemical quenching was perceived with increasing salinity concentration, with a maximum value of 0.85 recorded under 50 mM NaCl, showing variation compared with control conditions.

When plants experience elevated levels of salinity, it disrupts their cellular oxidation balance (**Figure 4**). In a current experiment, it is observed that POD and CAT actively returned to oxidative destruction in *Ae. tauschii* subjected to various salinity stress levels. The salinity stress had a minor impact on POD movement and did not show a significant outcome on leaf POD content. However, there was a notable decrease in CAT contents with increasing salinity levels in *Ae. tauschii*. Proline, an important component of plant proteins, is synthesized and accumulates in large quantities in response to abiotic stresses like high-salt and drought conditions. Our findings revealed a decrease in proline levels in *Ae. tauschii* as salinity stress increased. Conversely, under drought conditions, the Malondialdehyde (MDA) content significantly decreased in *Ae. tauschii*.

### 3.3 Effect of drought on different physiological and biochemical activities

**Figure 5:**
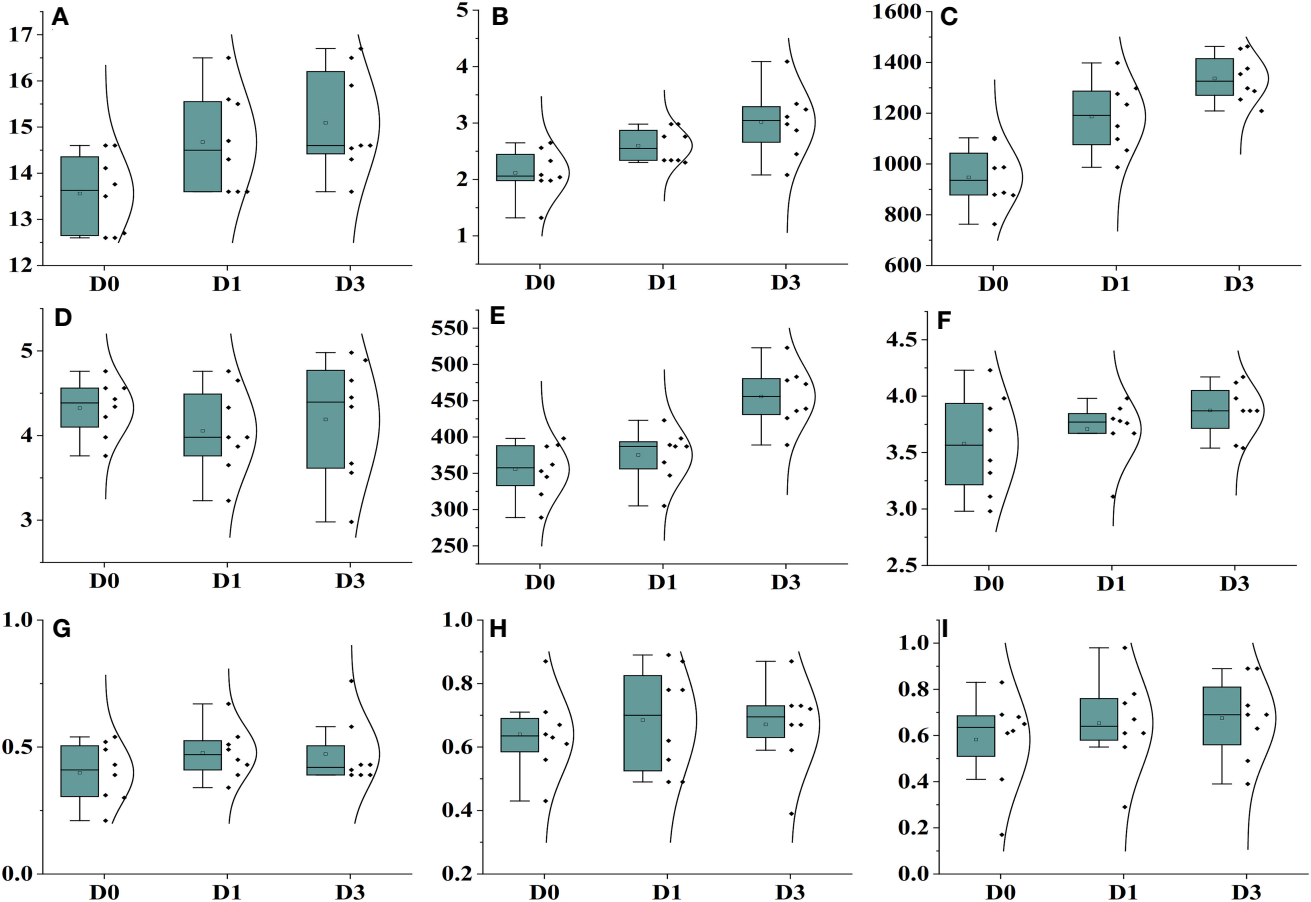
Effect of different drought stress levels on different physiological and biochemical parameters. **(A)** Plant height, **(B)** plant biomass, **(C)** POD, **(D)** MDA, **(E)** CAT, **(F)** proline, **(G)** FM, **(H)** YPQ, and **(I)** inh.

Drought stress exerted a significant influence on all traits, except for plant height, encompassing a range of physiological and biochemical parameters (**Figure 5**; **Supplementary Table 5**). The dry weight biomass allocation in *Ae. tauschii* was markedly impacted by varying levels of drought stress. Under induced drought conditions at 100 g L^−1^, the minimum and maximum values of dry weight biomass were recorded as 2.08 g and 4.09 g, respectively. In terms of plant height, the maximum height of 16.4 cm was observed under Polyethylene glycol (PEG)-induced drought stress (75 g L^−1^), whereas the minimum height of 11.3 cm was recorded under control drought stress, indicating a 21% reduction in plant height under the latter condition. Drought stress also had a significant impact on chlorophyll fluorescence (**Supplementary Table 3**). Fm′ exhibited a steady decrease as water availability declined. The maximum value of Fm′ was measured at 0.76 under 100 PEG, whereas the minimum value was 0.39. The quantum yield of PSII also displayed variation with water availability, with the maximum value of 0.84 observed under high PEG at 75 g L^−1^, and the minimum value was 0.19. Photochemical quenching also escalated to 0.98 under PEG at 100 g L^−1^ achieving a minimum of 0.43, whereas Inh.1 was 0.98, but it decreased to 0.29 under PEG at 75 g L^−1^.

When plants are exposed to high salinity levels, it disrupts the delicate balance of cellular oxidation in their organisms. Our study unveiled that the enzymes POD and CAT energetically respond to oxidative destruction in *Ae. tauschii* when subjected to various levels of drought stress levels. The treatment of drought stress had a minimal impact on the activity of POD and did not exhibit a significant effect on the content of leaf POD but showed some impact on CAT activities and also no impact on proline concentration but showed little variations. MDA content in *Ae. tauschii* decreased with increase drought stress levels.

### 3.4 Expression profile of salinity and drought tolerance genes

**Figure 6:**
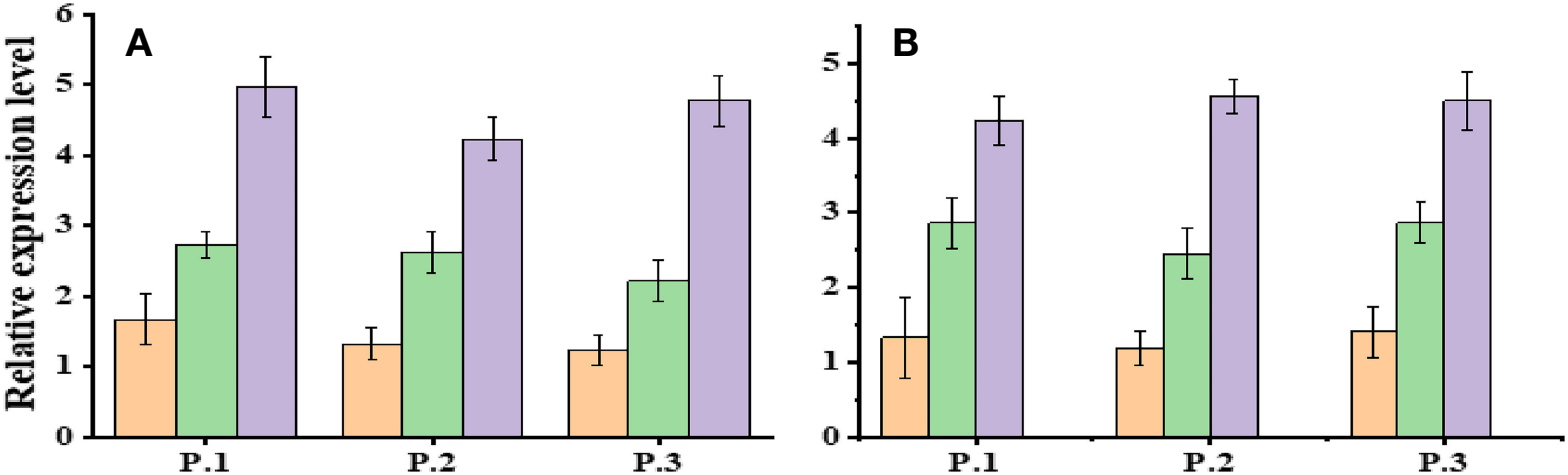
Relative expression levels of DREB.1 and DREB.2 in leaves of *Ae. tauschii* populations under drought conditions. Where’s, **(A)** DREB.1 and **(B)** DREB.2.

**Figure 7:**
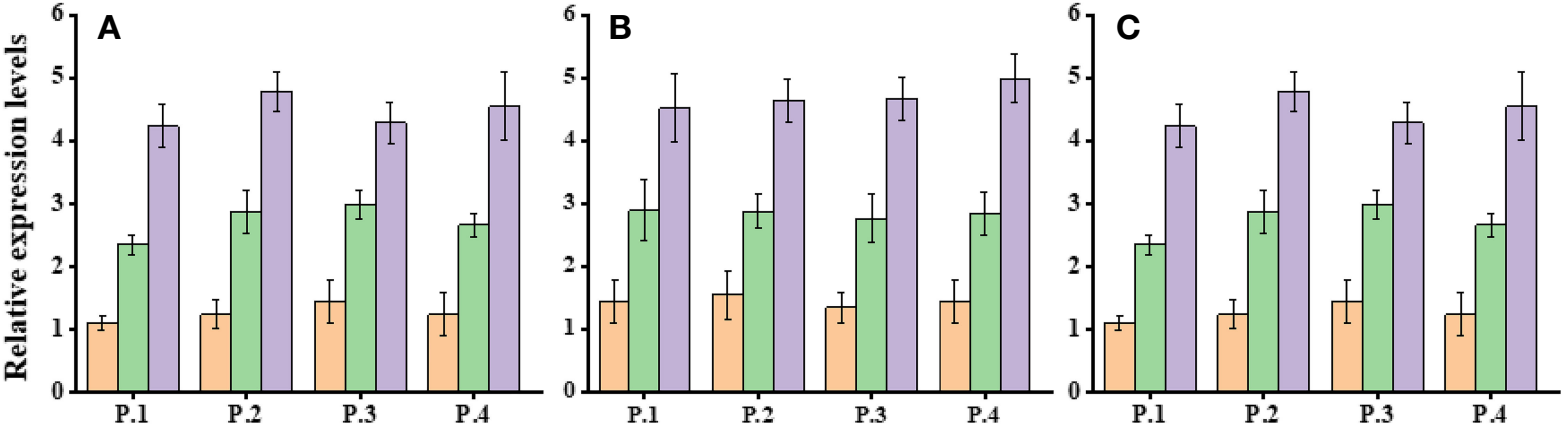
Relative expression levels of HKT.1, HKT.2, and HKT.3 in leaves of *Ae. tauschii* populations under salinity stress conditions. **(A)** HKT.1, **(B)** HKT.2 and **(C)** HKT.3.

Expression analysis of Dehydration responsive element-binding-1 (DREB-1) and DREB-2 was conducted in three populations of *Ae. tauschii* under control conditions (D1) and varying levels of drought stress (PEG-6000 of 75 g L^−1^ for D2 and D3). Significant changes in gene expression were observed across these populations in response to different drought stress levels (**Figure 6**). The expression profile of the DREB gene exhibited variability, with notably increased expression levels under drought stress conditions. Specifically, when exposed to PEG-6000 at a concentration of 75 g L^−1^ as a drought stress treatment, the expression levels of DREB were significantly higher compared with normal conditions. AeDREB.1 showed a 2.3-, 2.8-, 2.9-, and 3-fold increase in expression under PEG-6000 of 75 g L^−1^ compared with normal conditions. Under PEG-6000 of 100 g L^−1^, the expression profile exhibited a 4.5-, 5.4-, 4.6-, and 5.1-fold increase compared with normal conditions. Similar expression patterns were observed for AeDREB.2 under different drought conditions. Furthermore, we examined the expression levels of AeHKT1;1, AeHKT1;2, and AeHKT1;3 in leaves of four different populations of *Ae. tauschii* under control conditions (S1) and two salinity levels (S2, 50 mM NaCl; and S3, 100 mM NaCl). Real-time PCR analysis was performed to determine the relative expression patterns of these genes (**Figure 7**). The expression level of AeHKT1;4 exhibited high values in leaves (**Figures 4A, B**). AeHKT1;1 displayed a 2.1-, 2.3-, 2.5-, and 2.8-fold increase in expression under 50 mM NaCl compared with normal conditions. Under 100 mM NaCl, the expression profile showed a 4.1-, 4.2-, 4.4-, and 4.7-fold increase compared with normal conditions in the four different populations. Similar expression patterns were observed for AeHKT1;2 under different salinity conditions.

## 4 Discussion

Different physiological parameters, such as plant height, leaf count, leaf area, and tiller number, in *Ae. tauschii* are influenced by varying plant density and N levels. The impact of different N concentrations on this species has notable effects on photosynthetic activity, leading to increased biomass production. The photosynthetic activities of plants are closely tied to the availability of N, which, in turn, enhances their chlorophyll content and pushes their development and growth (Abdelsattar et al., 2023). Furthermore, increased N availability also enhances other physiological parameters such as leaf area ratio and tiller production in plants. However, variations in plant density have contrasting effects, leading to reduced biomass and height in *Ae. tauschii*. These kind cultural control activities also performed in many ways to know their behavior and mechanism and compared with different crops (Leal et al., 2023). Elevating the density of wheat plants resulted in a reduction in both the aboveground and belowground biomass of *Ae. tauschii*. However, augmenting N levels proved beneficial in increasing the biomass of various plant parts, including stems, leaves, and inflorescences. The addition of N has been observed to positively influence the biomass of various weed species found within crop fields at different growth stages, and this phenomenon attributed to the augmentation of key parameters such as leaf area, leaf area per plant, and plant height. The availability of N resources creates favorable conditions for weed growth, leading to an overall increase in biomass accumulation throughout the weed’s life cycle (Brooker et al., 2023). Because of high SPAD, plant height–to–leaf area ratio values in *Ae. tauschii* have a feature to compete with wheat crop for better N use efficiency (Masthigowda et al., 2023). During N applications with different plant density levels, Leaf weight ratio (LWR), SSL, and root shoot ratio (RSR) were increased with increasing N concentrations under different levels of density, some studies also reported that N enrichment also increased different physiological parameters of weeds including *Ae. Tauschii* (Zhang H. et al., 2023). The findings highlight that, under stressful conditions, *Ae. tauschii* exhibits an enhanced phenotypic plasticity characterized by increased nutrient absorption and a shift in resource allocation toward above-ground plant structures relative to underground components. This result emphasized that, under stress condition, phenotypic plasticity characteristic of *Ae. tauschii* increased, absorbed more nutrients, and increased upper plant parts than underground plant parts; thus LWR and SSL increased and RSR decreased. The application of Radiation Use Efficiency (RUE) is helpful to estimate crop growth, yield prediction before maturity, weed competition, and other production estimates (Asghar et al., 2023). Leaf area, crop surface exposure, and light are crucial for estimating crop yield. Adding N during the crop’s vegetative phase can enhance crop growth and leaf area development. However, weed competition, available N, and nutrient distribution within the plant affect N application. Adjusting plant density and nutrient supply is essential for understanding crop–weed interactions (Zhang Y. et al., 2023).

In our study, under high salinity conditions, lower biomass and higher Na^+^ values were recorded, indicating a potential adaptation through natural selection to salinity stress (Asghar et al., 2023). This phenomenon also elucidates why the majority of salinity-tolerant populations have evolved processes to flourish in saline conditions. These methods encompass osmotic modification, reduction of salinity levels through excretion and elimination, and the prevention of detrimental effects through compartmentalization (Lafond-Hudson and Sulman, 2023). A normal plant that possesses low Na^+^ intensity and high biomass demonstrates an enhanced tolerance. In addition, this study documented salt elimination in *Ae. Tauschii* (Melino and Tester, 2023). Salinity and drought stress exert detrimental effects on plant cell membrane systems. Several findings have provided evidence of the accumulation of MDA, CAT, POD, and proline contents are subjected to drought stress in plants. These compounds have an important role in retaining the equilibrium of the antioxidant system and alleviating cell damage induced by ROS. Antioxidant enzyme systems are pivotal in enhancing plant resistance to stress. CAT safeguards cell membranes from damage, POD eliminates excessive ROS, MDA serves as the effect of lipid peroxidation, and the deposit of proline signifies the plant’s ability to withstand drought conditions (Yan et al., 2023). In the current study, *Ae. tauschii* demonstrated noteworthy elevations in CAT, proline, and MDA contents within their leaves when exposed to salinity and drought stress. However, there was no significant alteration observed in POD. The levels of CAT, proline, and MDA in leaves exhibited a positive correlation with the intensity of the salinity and drought stress conditions (Wang et al., 2023). These findings indicate that *Ae. tauschii* employed mechanisms to alleviate oxidative injury by modulating the intensity of free proline and regulating the commotion of antioxidant activities in response to varying levels of salinity and drought stress (Chaouachi et al., 2023).

The gene responsible for salinity tolerance in wheat is situated at the distal region of chromosome 5AL and encodes a Na^+^ transporter from the HKT family. *Ae. tauschii*, the progenitor of the wheat crop with the DD genome, also carries a gene linked to salinity tolerance, and its locus associated with HKT genes plays a vital role in governing the transport of Na^+^ and K^+^ ions, thus maintaining their equilibrium (Banouh et al., 2023). Furthermore, the DREB gene acts as a crucial regulator within the network governing drought stress tolerance in different plants, such as *Triticum aestivum* L. and *Arabidopsis thaliana* [50]. Similarly, HKT1;4 showed a notable expression in the leaves, especially under high salinity stress levels. This indicates that HKT family genes play a vital role in facilitating the removal of Na^+^ ions from xylem cells in response to salinity stress (Abbas et al., 2021a). Moreover, our results demonstrated a substantial rise in Na^+^ accumulation in the leaves of *Ae. tauschii* when subjected to salinity stress. This stress condition notably upregulated the expression of HKT family genes, enabling the efficient exclusion of redundant Na^+^ from the plant roots and thereby relieving Na^+^ noxiousness. One of the key strategies employed by plants to cope with salinity stress is the sequestration of Na^+^ into vacuoles, aiming to decrease its concentration within the cytoplasm (Arzani and Ashraf, 2016). We designated the DREB to examine its pattern under drought conditions. DREBs, which are transcription factors, play a regulatory role in various abiotic stresses, including drought stress. Numerous studies have investigated the expression profiles of DREBs in different plant species. However, only a limited number of plant species have demonstrated variations in their expression profiles and their association with chlorophyll, proline, and sugar content in plants (Chowrasia et al., 2023).

In summary, drought stress substantially affected various traits in *Ae. tauschii*, encompassing physiological and biochemical parameters, except for plant height. Dry weight biomass allocation showed marked variations, with minimum and maximum values recorded under differing drought conditions. Moreover, the gene responsible for salinity tolerance encodes a Na^+^ transporter from the HKT family. *Ae. tauschii*, the wheat progenitor with the DD genome, carries a significant gene linked to salinity tolerance, controlled by the HKT gene locus governing Na^+^ and K^+^ transport. Likewise, the DREB gene emerges as a key regulator in the drought stress response across various plants. Analyzing DREB patterns under drought conditions further deepens our understanding of its role in abiotic stress regulation. Ultimately, this study enhances our grasp of how plants navigate challenging environments, offering new avenues for refined crop breeding strategies.

## Data availability statement

The original contributions presented in the study are included in the article/**Supplementary Material**. Further inquiries can be directed to the corresponding authors.

## Author contributions

AA and MS convinced the research idea and conduct research. AA and RH conducted research, and AS carried out formal analysis. AA and MS wrote the original draft of the manuscript. UZ, SA, and ATA provided technical expertise to strengthen the research concept. SA and ATA helped in the funding acquisition. PH and DD supervised whole study. All authors contributed to the article and approved the submitted version.
