# Corrigendum: Investigating the dynamic responses of *Aegilops tauschii* Coss. to salinity, drought, and nitrogen stress: a comprehensive study of competitive growth and biochemical and molecular pathways

**DOI:** 10.3389/fpls.2024.1415974

**Published:** 2024-04-22

**Authors:** Adeel Abbas, Rashida Hameed, Muhammad Saeed, Aitezaz A. A. Shahani, Ping Huang, Daolin Du, Usman Zulfiqar, Saud Alamri, Alanoud T. Alfagham

**Affiliations:** ^1^ Institute of Environment and Ecology, School of Environment and Safety Engineering, Jiangsu University, Zhenjiang, China; ^2^ Department of Weed Science and Botany, The University of Agriculture, Peshawar, Pakistan; ^3^ Key Laboratory of Crop Sciences and Plant Breeding Genetics, College of Agriculture, Yanbian University, Yanji, Jilin, China; ^4^ Department of Agronomy, Faculty of Agriculture and Environment, The Islamia University of Bahawalpur, Bahawalpur, Pakistan; ^5^ Department of Botany and Microbiology, College of Science, King Saud University, Riyadh, Saudi Arabia

**Keywords:** abiotic stress, *Aegilops tauschii* Coss, gene expression, physio-chemical properties, mechanism

**Error in Author List**

In the published article, there was an error in the author list, and author Rashida Hameed was erroneously excluded as corresponding author. The corrected author list appears below.

**Adeel Abbas^1^** ^†^ **, Rashida Hameed^1^** ^*^ **^†^, Muhammad Saeed^2^** ^†^ **, Aitezaz A.A. Shahani^3^** ^†^ , Ping Huang^1*^, Daolin Du^1*^, Usman Zulfiqar^4^, Saud Alamri^5^, Alanoud T. Alfagham^5^

1Institute of Environment and Ecology, School of Environment and Safety Engineering, Jiangsu University, Zhenjiang, China

2Department of Weed Science and Botany, The University of Agriculture, Peshawar, Pakistan

3Key Laboratory of Crop Sciences and Plant Breeding Genetics, College of Agriculture, Yanbian University, Yanji, Jilin, China

4Department of Agronomy, Faculty of Agriculture and Environment, The Islamia University of Bahawalpur, Bahawalpur, Pakistan

5Department of Botany and Microbiology, College of Science, King Saud University, Riyadh, Saudi Arabia

†These authors have contributed equally to this work

* Correspondence: Ping Huang, huangjiehp@ujs.edu.cn; Daolin Du, daolindu@163.com; Rashida Hameed, hrashda@ymail.com

The authors apologize for this error and state that this does not change the scientific conclusions of the article in any way. The original article has been updated.

