# Supplementary material for: Investigating the dynamic responses of Aegilops tauschii Coss. to salinity, drought, and nitrogen stress: a comprehensive study of competitive growth and biochemical and molecular pathways
Source: Front Plant Sci. 2023 Sep 6;14:1238704. doi: 10.3389/fpls.2023.1238704 (PMC10511890; doi:10.3389/fpls.2023.1238704)
Supplement: Supplementary file 1 [file DataSheet_1.pdf]

Supplementary Material

Investigating the Dynamic Responses of *Aegilops tauschii* Coss to Salinity, Drought, and Nitrogen Stress: A Comprehensive Study of Competitive Growth, Biochemical and Molecular Pathways.

Adeel abbas[1], Rashida Hameed[1], Muhammad Saeed[2], Aitezaz A.A. Shahani[3], Ping Huang[1]*, Daolin Du[1], Usman Zulfiqar[4], Saud Alamri[5], Alanoud T. Alfagham[5]

*** Correspondence:** huangjiehp@ujs.edu.cn, daolindu@163.com

Table 1. Primer details for salinity and drought stress related genes.

Gene Name	Gene symbol	Forword Primer (5'->3'))	Reverse Primer (5'->3'))
Actin	LOC109759322	TTGCCTTGGATTATGAACA	GATGGCTGGAACAGAACTT
HKT1.1	LOC123046507	CACGGCTCTGTCACAACTCT	TTAGAAGGGGCTTGCCTGAC
HKT1.2	LOC109768841	AGTTTCGCACCCTCCAAGTT	TGTGTTCTGTGATGCCCCTC
HKT1.3	LOC123148632	ATGTAAGTTCCGCGCAGTCA	ACCCTGTAACTGTAAGCGCC
DREB.1	LOC125530935	GTCCTAGAGGACAAGGGCAC	GTCATGCGGAGCAATAGGGA
DREB.2	AT5G67190	ATAGAAAACCGCCGTGTCGT	AATACCACCTGCAAGCTCCC

Table 2. The effect of different wheat planting densities and nitrogen levels on different parameter of wheat crop.

	Shoot weight	Root weight	Biomass	Root shoot ratio	Tillers per plant
**110**	34.58	15.95	50.53	0.48	26.14
**220**	42.32	14.34	56.67	0.34	33.59
**440**	47.84	18.99	66.85	0.41	41.25
**S.E.D**	1.82	1.91	2.12	0.11	2.02
P	<0.001	<0.001	<0.001	0.002	<0.001
**Nitrogen levels**					
**0**	16.20	7.69	23.90	0.47	24.16
**60**	34.95	15.89	50.83	0.46	26.99
**120**	50.90	20.01	70.90	0.40	35.86
**180**	59.07	22.55	81.63	0.38	47.25
**S.E.D**	2.01	1.85	1.79	0.08	2.04
P	<0.001	<0.001	<0.001	0.002	<0.001

Table 3. Minimum, maximum and mean value of different photosynthesis parameters under different salinity levels.

	Fm	Y(NPQ)	NPQ1	Inh.1	Y(II)	Plant height	Plant biomass	POD	MDA	CAT	Proline
**S0**											
Mini.	0.181	0.58	0.39	0.13	0.34	11.4	1.65	773	3.56	311	3.11
Maxi.	0.42	0.83	0.98	0.75	0.82	15.50	1.99	943	3.11	411	4.23
Mean.	0.28	0.64	0.67	0.51	0.62	13.20	1.86	860.63	3.78	377	3.72
**S1**											
Mini.	0.15	0.43	0.53	0.23	0.23	12	1.29	987	2.11	342	2.54
Maxi.	0.51	0.93	0.85	0.84	0.87	16.5	1.99	1202	2.98	411	3.98
Mean.	0.35	0.64	0.62	0.57	0.47	14.36	1.86	1107	2.65	377	3.26
**S2**											
Mini.	0.16	0.39	0.34	0.34	0.34	12	1.54	1132	3.13	413	2.65
Maxi.	0.54	0.98	0.83	0.92	0.83	15.8	2.72	1365	4.23	543	3.78
Mean.	0.33	0.68	0.59	0.66	0.49	13.98	2.04	1254.12	3.67	449.25	3.44
**S3**											
Mini.	0.19	0.40	0.48	0.21	0.18	10	1.55	1276	2.54	219	2.76
Maxi.	0.47	0.83	0.87	0.82	0.53	15	2.44	1487	4.11	365	4.11
Mean.	0.29	0.65	0.69	0.63	0.39	12.63	2.05	1363.5	3.01	301.13	3.59

Table 4. Minimum, maximum, and mean value of different salinity traits under different salinity levels.

Treatment	50 mM NaCl			100 mM NaCl			200 mM NaCl		
	Na^+	K^+	Na^+/K^+	Na^+	K^+	Na^+/K^+	Na^+	K^+	Na^+/K^+
**S_0**									
**Mini.**	23.45	34.56	0.61	33.56	54.56	0.62	67.76	54.56	1.13
**Maxi.**	43.56	51.54	1.77	56.76	69.87	1.74	101.54	69.87	1.50
**Mean.**	32.02	45.06	0.95	45.25	63.67	1.31	81.86	63.67	1.29
**S_1**									
**Mini.**	87.90	101.54	0.67	101.34	156.54	0.54	165.76	156.54	0.99
**Maxi.**	134.54	178.65	1.47	154.65	211.42	1.61	287.65	211.43	1.45
**Mean.**	105.97	137.81	0.96	128.74	179.13	1.25	218.92	179.13	1.22
**S_2**									
**Mini.**	87.89	123.54	0.54	101.54	165.87	0.78	189.98	165.87	1.03
**Maxi.**	123.54	187.65	1.89	176.65	234.43	1.78	265.65	234.42	1.42
**Mean.**	103.30	150.89	0.97	148.90	190.27	1.24	223.89	190.27	1.18
**S3**									
**Mini.**	98.76	106.76	0.56	145.76	156.98	0.82	198,76	156.54	1.00
**Maxi.**	139.6	176.76	1.32	167.76	245.54	1.57	298.76	245.54	1.35
**Mean.**	117.01	150.56	0.92	158.33	193.69	1.18	228.64	193.69	1.19

Table 5. Minimum, maximum, and mean value of different photosynthesis and, biochemical parameters under different drought stress levels.

Drought levels	Fm	Y(NPQ)	NPQ1	Inh.1	Y(II)	Plant height	Plant biomass	POD	MDA	CAT	Proline
**D1**											
Mini.	0.21	0.43	0.45	0.17	0.23	12.6	1.32	763	3.76	289	2.98
Maxi.	0.54	0.64	0.89	0.83	0.92	14.6	2.65	1103	4.76	398	4.23
Mean.	0.39	0.83	0.70	0.58	0.59	13.55	2.11	947	4.32	355	3.58
**D2**											
Mini.	0.34	0.49	0.59	0.29	0.19	14.6	2.3	987	3.23	305	3.11
Maxi.	0.67	0.89	0.78	0.65	0.84	16.5	2.98	1398	4.76	423	3.98
Mean.	0.47	0.68	0.71	0.98	0.55	14.67	2.6	1186	4.05	375	3.70
**D3**											
Mini.	0.39	0.39	0.43	0.39	0.29	13.6	2.08	1209	2.98	389	3.54
Maxi.	0.76	0.67	0.66	0.67	0.55	16.70	4.09	1463	4.98	523	4.17
Mean.	0.47	0.87	0.98	0.89	0.83	15.09	3.02	1336.87	4.19	455.87	3.87
